# Novel methods for wind speeds prediction across multiple locations

**DOI:** 10.1038/s41598-022-24061-4

**Published:** 2022-11-15

**Authors:** Oleg Gaidai, Jingxiang Xu, Ping Yan, Yihan Xing, Yu Wu, Fuxi Zhang

**Affiliations:** 1 Shanghai Engineering Research Center of Marine Renewable Energy, College of Engineering Science and Technology, Shanghai Ocean University, Shanghai, China; 2 University of Stavanger, Stavanger, Norway

**Keywords:** Environmental sciences, Engineering, Mathematics and computing

## Abstract

This article provides two unique methodologies that may be coupled to study the dependability of multidimensional nonlinear dynamic systems. First, the structural reliability approach is well suited for multidimensional environmental and structural reactions and is either measured or numerically simulated over sufficient time, yielding lengthy ergodic time series. Second, a unique approach to predicting extreme values has technical and environmental implications. In the event of measurable environmental loads, it is also feasible to calculate the probability of system failure, as shown in this research. In addition, traditional probability approaches for time series cannot cope effectively with the system's high dimensionality and cross-correlation across dimensions. It is common knowledge that wind speeds represent a complex, nonlinear, multidimensional, and cross-correlated dynamic environmental system that is always difficult to analyze. Additionally, global warming is a significant element influencing ocean waves throughout time. This section aims to demonstrate the efficacy of the previously mentioned technique by applying a novel method to the Norwegian offshore data set for the greatest daily wind cast speeds in the vicinity of the Landvik wind station. This study aims to evaluate the state-of-the-art approach for extracting essential information about the extreme reaction from observed time histories. The approach provided in this research enables the simple and efficient prediction of failure probability for the whole nonlinear multidimensional dynamic system.

## Introduction

Accurate estimations of wind resource and operational conditions are beneficial for the cost-effective growth of the wind energy sector. Extreme value analysis is of relevance in a variety of domains since it deals with statistical inference on extreme values. Environmental extremes (such as river flow, wind speeds, temperature, and rainfall) and engineering are two of the key areas of concentration (e.g., structural reliability and strength of materials).

In general, utilizing traditional theoretical reliability methods to calculate the dependability of practical environmental systems may be rather difficult. The latter is often brought on by a large number of system freedoms and random variables that control the environment system. In theory, it is possible to estimate the dependability of complex environmental systems in a straightforward manner by using either a sufficient number of measurements or direct Monte Carlo simulations. For many complicated environmental systems, the experimental or computational costs may be prohibitive. Authors have developed a novel reliability method for environmental systems with the goal of lowering measurement or computational costs.

## Methods

It is challenging to estimate structural system reliability using classic engineering methods^1,2^. The latter is usually due to a large number of degrees of system freedom and random variables governing dynamic systems. The reliability of complex structural systems can be straightforwardly estimated either by having enough measurements or by direct numerical Monte Carlo simulations^3–11^. However, experimental and computational often are unaffordable for many complex engineering dynamic systems. Authors have introduced a novel reliability method for structural systems aiming to reduce measurement or numerical computational costs.

Ocean waves are usually assumed to be a random ergodic process (stationary and homogenous). Consider MDOF (multi-degree of freedom) offshore structure subjected to ergodic environmental loadings, for example, originating from surrounding waves and wind. MDOF structural response vector process ${\varvec{R}}\left(t\right)=\left(X\left(t\right), Y\left(t\right), Z\left(t\right), \ldots \right)$, that has been either measured or simulated over a long enough time interval $(0,T)$. Unidimensional response process maxima over the entire time span $(0,T)$ denoted as ${X}_{T}^{\mathrm{max}}=\underset{0\le t\le T}{\mathrm{max}}X\left(t\right)$, ${Y}_{T}^{\mathrm{max}}=\underset{0\le t\le T}{\mathrm{max}}Y\left(t\right)$, ${Z}_{T}^{\mathrm{max}}=\underset{0\le t\le T}{\mathrm{max}}Z\left(t\right), \ldots$.

Let ${X}_{1},\ldots ,{X}_{{N}_{X}}$ represent local maxima of the time-dependent process $X(t)$ at discrete monotonically rising time moments ${t}_{1}^{X}< \cdots <{t}_{{N}_{X}}^{X}$ in $(0,T)$. Similar definitions may be provided for the additional MDOF response components $Y\left(t\right), Z\left(t\right), \ldots$ using ${Y}_{1}, \ldots ,{Y}_{{N}_{Y}}; {Z}_{1}, \ldots ,{Z}_{{N}_{Z}}$,…. All MDOF ${\varvec{R}}\left(t\right)$ components and their unidimensional maxima are considered to be bigger than zero in this investigation^12^.

The objective is to determine the MDOF system failure probability, also known as the exceedance probability

1 $$1-P=\mathrm{Prob}({X}_{T}^{\mathrm{max}}>{\eta }_{X} \cup {Y}_{T}^{\mathrm{max}}>{\eta }_{Y} \cup {Z}_{T}^{\mathrm{max}}>{\eta }_{Z} \cup \ldots )$$

with $P=\underset{\left(0, 0, 0, \ldots \right)}{\overset{\left({\eta }_{X}, {\eta }_{Y}, {\eta }_{Z }, \ldots \right)}{\iiint }}{p}_{{X}_{T}^{\mathrm{max}}, { Y}_{T}^{\mathrm{max}}, { Z}_{T}^{\mathrm{max}} , \ldots }\left({X}_{T}^{\mathrm{max}}, {Y}_{T}^{\mathrm{max}},{ Z}_{T}^{\mathrm{max}}, \ldots \right)d{X}_{T}^{\mathrm{max}}d{Y}_{{N}_{Y}}^{\mathrm{max}}d{Z}_{{N}_{z}}^{\mathrm{max}}\ldots$ being the non-exceedance probability for critical unidimensional response values ${\eta }_{X}$, ${\eta }_{Y}$, ${\eta }_{Z}$,…; with $\cup$ denoting logical unity operation; and ${p}_{{X}_{T}^{\mathrm{max}}, { Y}_{T}^{\mathrm{max}}, { Z}_{T}^{\mathrm{max}} , \ldots }$ denoting the global maxima joint probability density over the time period $(0,T)$. In reality, it is difficult to directly estimate the joint probability distribution due to its large dimensionality and the data collection limits.

**Figure 1:**
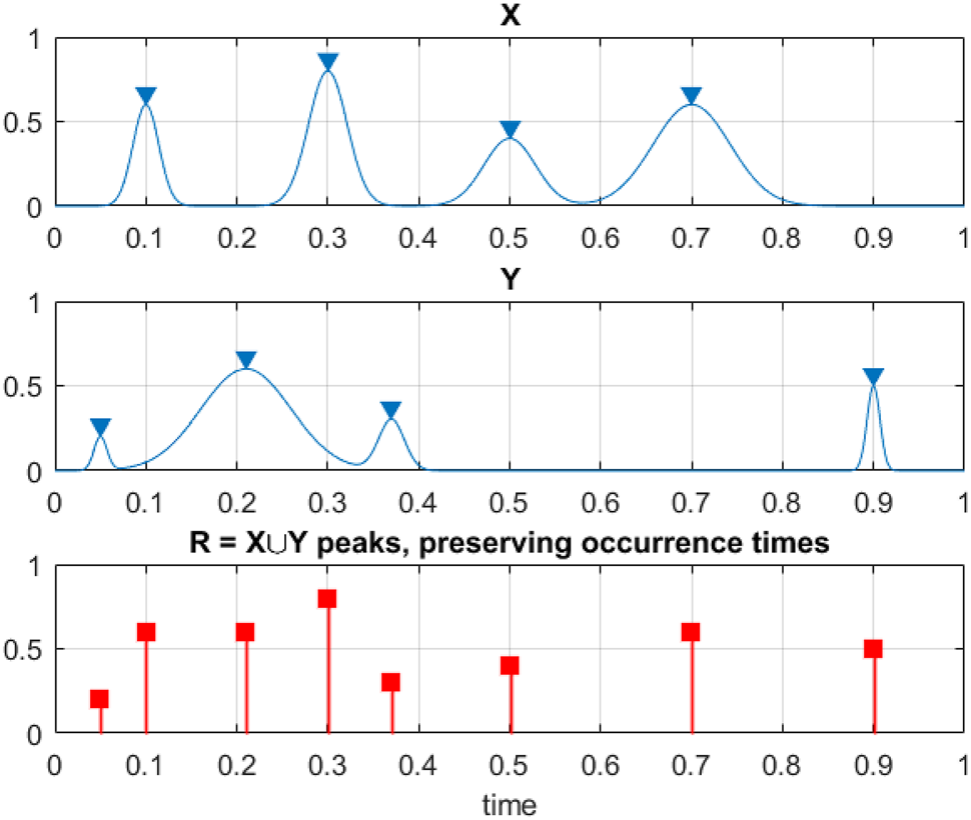
Illustration of how two exemplary processes, X and Y, are combined into a new synthetic vector ${\varvec{R}}\left(t\right)$.

The moment in time when either $X\left(t\right)$ exceeds ${\eta }_{X}$, or $Y\left(t\right)$ exceeds ${\eta }_{Y}$, or $Z\left(t\right)$ exceeds ${\eta }_{Z}$, and so on, the dynamic system is regarded as efficiently failed. Pre-defined failure levels ${\eta }_{X}$, ${\eta }_{Y}$, ${\eta }_{Z}$,…are individual for every unidimensional response component of ${\varvec{R}}\left(t\right)$. ${X}_{{N}_{X}}^{\mathrm{max}}=\mathrm{max }\{{X}_{j}\hspace{0.17em};j=1,\ldots ,{N}_{X}\}={X}_{T}^{\mathrm{max}}$, ${Y}_{{N}_{Y}}^{\mathrm{max}}=\mathrm{max }\{{Y}_{j}\hspace{0.17em};j=1,\ldots ,{N}_{Y}\}={Y}_{T}^{\mathrm{max}}$,${Z}_{{N}_{z}}^{\mathrm{max}}=\mathrm{max }\{{Z}_{j}\hspace{0.17em};j=1,\ldots ,{N}_{Z}\}={Z}_{T}^{\mathrm{max}}$,…. , see Fig. 1.

The next step is to sort time instants unidimensional response component maxima $\left[{t}_{1}^{X}<\cdots <{t}_{{N}_{X}}^{X}; {t}_{1}^{Y}<\cdots <{t}_{{N}_{Y}}^{Y}; {t}_{1}^{Z}<\cdots <{t}_{{N}_{Z}}^{Z}\right]$ in non-decreasing temporal order into one single merged time series vector ${t}_{1}\le \cdots \le {t}_{N}$, with ${t}_{N}=\mathrm{max }\{{t}_{{N}_{X}}^{X}, {t}_{{N}_{Y}}^{Y}, { t}_{{N}_{Z}}^{Z}, \ldots \}$, $N={N}_{X}+{N}_{Y}+{ N}_{Z}+ \cdots$. With ${t}_{j}$ representing unidimensional response component maxima of one of the MDOF structural response components. So having ${\varvec{R}}\left(t\right)$, one must concurrently and continuously screen for each unidimensional response component temporal maxima, noting its exceedances of MDOF limit vector $\left({\eta }_{X}, {\eta }_{Y}, {\eta }_{Z},\ldots\right)$ in any of its unidimensional response components $X, Y, Z, \ldots$ Each unidimensional response component maxima are then combined into a single temporal non-decreasing vector $\overrightarrow{R}=\left({R}_{1}, {R}_{2}, \ldots ,{R}_{N}\right)$ based on the merged time vector ${t}_{1}\le \cdots \le {t}_{N}$. Finally, the unified MDOF limit vector $\left({\eta }_{1}, \ldots ,{\eta }_{N}\right)$ is now introduced with each component ${\eta }_{j}$ being either ${\eta }_{X}$, ${\eta }_{Y}$ or ${\eta }_{Z}$ …, depending on which of $X\left(t\right)$ or $Y\left(t\right)$, or $Z\left(t\right)$ … corresponds to the current unidimensional response component maxima with the running index $j$, see^12–16^.

Scaling parameter $0<\lambda \le 1$ is now added to artificially lower limit values for all unidimensional response components, namely the new MDOF limit vector $\left({\eta }_{X}^{\lambda },{ \eta }_{Y}^{\lambda }, {\eta }_{z}^{\lambda },\ldots\right)$ with ${\eta }_{X}^{\lambda }\equiv \lambda {\cdot \eta }_{X}$, $\equiv \lambda {\cdot \eta }_{Y}$, ${\eta }_{z}^{\lambda }\equiv \lambda {\cdot \eta }_{Z}$, … is now introduced, see^17^. The unified MDOF limit vector $\left({\eta }_{1}^{\lambda }, \ldots ,{\eta }_{N}^{\lambda }\right)$ has each component ${\eta }_{j}^{\lambda }$ as either ${\eta }_{X}^{\lambda }$, ${\eta }_{Y}^{\lambda }$ or ${\eta }_{z}^{\lambda }$ …. The latter specifies the probability distribution function $P\left(\lambda \right)$ as a function of artificial parameter $\lambda$, note that $P\equiv P\left(1\right)$ from Eq. (1). The non-exceedance probability function $P\left(\lambda \right)$ may be expressed as follows

2 $$\begin{aligned} P\left(\lambda \right) & =\mathrm{Prob}\{{R}_{N}\le {\eta }_{N}^{\lambda }, \ldots ,{R}_{1}\le {\eta }_{1}^{\lambda }\} \\ & =\mathrm{Prob}\left\{{R}_{N}\le {\eta }_{N}^{\lambda } \right| {R}_{N-1}\le {\eta }_{N-1}^{\lambda }, \ldots ,{R}_{1}\le {\eta }_{1}^{\lambda }\}\cdot \mathrm{Prob}\{{R}_{N-1}\le {\eta }_{N-1}^{\lambda }, \ldots ,{R}_{1}\le {\eta }_{1}^{\lambda }\} \\ & =\prod_{j=2}^{N}\mathrm{Prob}\{{R}_{j}\le {\eta }_{j}^{\lambda } |{ R}_{j-1}\le {\eta }_{1j-}^{\lambda }, \ldots ,{R}_{1}\le {\eta }_{1}^{\lambda }\}\cdot \mathrm{Prob}({R}_{1}\le {\eta }_{1}^{\lambda }) \end{aligned}$$

The cascade of approximations based on conditioning is described. In practice, dependencies between adjacent local maxima ${R}_{j}$ are not insignificant; hence the following one step (defined as conditioning level $k=1$) memory approximation may be implemented

3 $$\mathrm{Prob}\{{R}_{j}\le {\eta }_{j}^{\lambda } |{ R}_{j-1}\le {\eta }_{j-1}^{\lambda }, \ldots ,{R}_{1}\le {\eta }_{1}^{\lambda }\}\approx \mathrm{Prob}\{{R}_{j}\le {\eta }_{j}^{\lambda } |{ R}_{j-1}\le {\eta }_{j-1}^{\lambda }$$

for $2\le j\le N$ (defined as conditioning level $k=2$). Then Eq. (3) can be further developed as

4 $$\mathrm{Prob}\{{R}_{j}\le {\eta }_{j}^{\lambda } |{ R}_{j-1}\le {\eta }_{j-1}^{\lambda }, \ldots ,{R}_{1}\le {\eta }_{1}^{\lambda }\}\approx \mathrm{Prob}\{{R}_{j}\le {\eta }_{j}^{\lambda } |{ R}_{j-1}\le {\eta }_{j-1}^{\lambda }, {R}_{j-2}\le {\eta }_{j-2}^{\lambda }\}$$

with $3\le j\le N$ (defined as level $k=3$). Equation (4) presents subsequent refinements of local consecutive maxima independence assumption. The latter reflects the statistical dependence between neighbouring local maxima with ever-increasing accuracy. As the MDOF response process ${\varvec{R}}\left(t\right)$ was assumed ergodic, the probability ${p}_{k}\left(\lambda \right):=\mathrm{Prob}\left\{{R}_{j}>{\eta }_{j}^{\lambda } \right| {R}_{j-1}\le {\eta }_{j-1}^{\lambda }, {R}_{j-k+1}\le {\eta }_{j-k+1}^{\lambda }\}$ for $j\ge k$ is independent of $j$ and entirely on the conditioning level $k$

5 $${P}_{k}(\lambda )\approx \mathrm{exp }(-{N\cdot p}_{k}\left(\lambda \right))\hspace{0.17em} ,\quad k\ge 1.$$

that Eq. (5) is similar to the mean up-crossing rate formula for the probability of exceedance^4^. There is gradual convergence with respect to the conditioning parameter $k$

6 $$P=\underset{k\to \infty }{\mathrm{lim}}{P}_{k}(1);\quad p\left(\lambda \right)=\underset{k\to \infty }{\mathrm{lim}}{p}_{k}\left(\lambda \right)$$

Note that Eq. (5) $k=1$ case corresponds to the non-exceedance probability relationship with the mean up-crossing rate function

7 $$P\left( \lambda \right){\text{~}} \approx {\text{exp~}}( - \nu ^{ + } \left( \lambda \right)\,T);~\nu ^{ + } \left( \lambda \right) = \int_{0}^{\infty } {\zeta p_{{R\dot{R}}} \left( {\lambda ,\zeta } \right)d\zeta }$$

with ${\nu }^{+}(\lambda )$ being the mean up-crossing rate of the response level $\lambda$ for the above assembled non-dimensional vector $R\left(t\right)$ assembled from the scaled MDOF system response $\left(\frac{X}{{\eta }_{X}}, \frac{Y}{{\eta }_{Y}}, \frac{Z}{{\eta }_{Z}}, \ldots \right)$. The mean up-crossing rate from Rice's formula given in Eq. (7) with ${p}_{R\dot{R}}$ being the joint probability density for $\left(R, \dot{R}\right)$ with $\dot{R}$ being time derivative $R{^{\prime}}\left(t\right)$, see^18^. Equation (7) relies on the Poisson assumption, that is, up-crossing events of high $\lambda$ levels (in this paper it is $\lambda \ge 1$) can be assumed to be independent.

In the preceding, the assumption of ergodicity was used. For non-stationary cases, the illustration will be done for offshore engineering and naval architecture areas of engineering applications. Given, for example, the scattered diagram of $m=1, \ldots ,M$ environmental states, each short-term environmental state has the probability ${q}_{m}$, so that $\sum_{m=1}^{M}{q}_{m}=1$. Next, the long-term equation can be introduced

8 $${p}_{k}(\lambda )\equiv \sum_{m=1}^{M}{p}_{k}(\lambda ,m){q}_{m}$$

with ${p}_{k}(\lambda ,m)$ being the similar function as in Eq. (6), but corresponding to a short-term environmental state with the number $m$.

Next, the Naess-Gaidai (NG) approach^11^ is presented briefly. The above-described ${p}_{k}(\lambda )$ functions are often tail-regular, i.e. for extreme values of $\lambda$ approaching extreme level $1$. More specifically, for $\lambda \ge {\lambda }_{0}$, the distribution tail behaves like ${\text{exp}}\left\{-{\left(a\lambda +b\right)}^{c}+d\right\}$ with $a, b, c, d$ being fitted constants for appropriate tail cut-on ${\lambda }_{0}$ value. NG method assumes

9 $${p}_{k}(\lambda )\approx {\text{exp}}\left\{-{\left({a}_{k}\lambda +{b}_{k}\right)}^{{c}_{k}}+{d}_{k}\right\}, \lambda \ge {\lambda }_{0}$$

When ${\text{ln}}\left\{{\text{ln}}\left({p}_{k}(\lambda )\right)-{d}_{k}\right\}$ is plotted against ${\text{ln}}\left({a}_{k}\lambda +{b}_{k}\right)$, almost linear tail behaviour is often seen. The logarithmic level was optimized by minimizing the error function *F* with regard to its four parameters ${a}_{k}, {b}_{k}, {c}_{k},{p}_{k},{q}_{k}$

10 $$F\left( {a_{k} ,~b_{k} ,~c_{k} ,p_{k} ,q_{k} } \right) = \int_{{\lambda _{0} }}^{{\lambda _{1} }} {\omega \left( \lambda \right)} \left\{ {{\text{ln}}\left( {p_{k} \left( \lambda \right)} \right) - d_{k} + \left( {a_{k} \lambda + b_{k} } \right)^{{c_{k} }} } \right\}^{2} d\lambda ,~~~\lambda \ge \lambda _{0}$$

with ${\lambda }_{1}$ being an appropriately selected distribution tail cut-off number for which the breadth of the confidence interval (CI) is acceptable. The optimal parameters ${a}_{k}, {b}_{k}, {c}_{k},{p}_{k},{q}_{k}$ may be calculated using the sequential quadratic programming (SQP) approach implemented in NAG Numerical Library^19^.

### Novel extrapolation method

Accurate prediction of extreme values is a typical and demanding engineering dependability challenge, particularly when data is insufficient. Consequently, the development of unique, efficient, and precise extrapolation methods is of enormous practical value.

Consider a stationary stochastic process $X\left(t\right)$, either simulated or measured over a given time interval $0\le t\le T$, and which is represented as a sum of two independent stationary processes ${X}_{1}\left(t\right)$ and ${X}_{2}\left(t\right)$, namely

11 $$X\left(t\right)={X}_{1}\left(t\right)+{X}_{2}\left(t\right)$$

Note that this study seeks to provide a generic technique applicable to predicting extreme values for a broad range of loads and reactions for various vessels and offshore constructions. For the process of interest $X\left(t\right)$, marginal PDF (probability density function) ${p}_{X}$ may be obtained in two ways:

A. By directly extracting ${p}_{X}^{A}$ from the provided data set, i.e. time series $X\left(t\right)$,
B. By separately extracting PDFs from the process components ${X}_{1}\left(t\right)$ and ${X}_{2}\left(t\right)$, namely ${p}_{{X}_{1}}$ and ${p}_{{X}_{2}}$, then using convolution ${p}_{X}^{B}={\text{conv}}\left({ p}_{{X}_{1}},{ p}_{{X}_{2}}\right)$.

Both ${p}_{X}^{A}$ and ${p}_{X}^{B}$ are approximations of the PDF goal, ${p}_{X}^{A}$ is simpler to implement, however B) would yield a more precise estimate of the desired PDF, ${p}_{X}$. Convolution permits extrapolation of the directly derived empirical PDF, ${p}_{X}^{A}$, without supposing any particular extrapolation functional class, such as generalized extreme value distributions (GEV), required to extrapolate distribution tail towards the design low probability level of interest. The majority of current extrapolation techniques, which are frequently used in engineering practice, depending on the assumption of certain extrapolation functional classes, e.g.^3–9^.

The two independent component representation provided by Eq. (11) is seldom accessible; consequently, one may seek artificial methods to estimate, ${p}_{{X}_{1}}$ and ${p}_{{X}_{2}}$, or in the most straightforward situation, discover two identically distributed process components, ${X}_{1}\left(t\right)$ and ${X}_{2}\left(t\right)$ with ${p}_{{X}_{1}}={ p}_{{X}_{2}}$. This work focuses on the latter possibility, in which processes, ${X}_{1}\left(t\right)$ and ${X}_{2}\left(t\right)$ are dispersed equally. Given the directly estimated distribution, ${p}_{X}$, as in option A, the present study's objective would be to identify the component distribution, ${p}_{{X}_{1}}$, such that

12 $${ p}_{X}={\text{conv}}\left({ p}_{{X}_{1}},{ p}_{{X}_{1}}\right)$$

therefore, limiting the scope of this research to a deconvolution situation. To illustrate the latter concept of how to estimate the unknown distribution, ${p}_{{X}_{1}}$, reliably and then enhance (or extrapolate) the supplied empirical distribution, ${p}_{X}$.

### Discrete convolution

This section provides a concise overview of commonly held knowledge surrounding the discrete convolution of two vectors. The convolution of two vectors, ${\varvec{u}}$ and ${\varvec{v}}$, illustrates the vector component overlap as ${\varvec{v}}$ glides over ${\varvec{u}}$. Convolution corresponds algebraically to multiplying polynomials whose coefficients are the elements of ${\varvec{u}}$ and ${\varvec{v}}$.

Let $m={\text{length}}\left({\varvec{u}}\right)$ and $n={\text{length}}\left({\varvec{v}}\right)$. Then ${\varvec{w}}$ is the vector of length $m+n-1$, whose $k$-th element is

13 $$w\left(k\right)=\sum_{j=1}^{m}u\left(j\right)v\left(k-j+1\right)$$

The sum is over all the values of $j$ that lead to legal subscripts for $u\left(j\right)$ and $v\left(k-j+1\right)$, specifically $j={\text{max}}\left(1,k+1-n\right):1:\mathrm{min}\left(k,m\right)$. When $m=n$, as will be the case in this paper, the latter gives

14 $$\begin{array}{*{20}c} {w\left(1\right)=u\left(1\right)\cdot v\left(1\right)} \\ {w\left(2\right)=u\left(1\right)\cdot v\left(2\right)+u\left(2\right)\cdot v\left(1\right)} \\ {w\left(3\right)=u\left(1\right)\cdot v\left(3\right)+u\left(2\right)\cdot v\left(2\right)+u\left(3\right)\cdot v\left(1\right)} \\ \cdots \\ { w\left(n\right)=u\left(1\right)\cdot v\left(n\right)+u\left(2\right)\cdot v\left(n-1\right)+\cdots +u\left(n\right)\cdot v\left(1\right) } \\ \cdots \\ { w\left(2n-1\right)=u\left(n\right)\cdot v\left(n\right) } \\ \end{array}$$

Having obtained ${\varvec{u}}={\varvec{v}}=\left(u\left(1\right), \ldots ,u\left(n\right)\right)$, one may progressively acquire ${\varvec{w}}$**-**components $w\left(n+1\right),\ldots,w\left(2n-1\right)$, as the index ranges from $n+1$ to $2n-1$. Clearly, the latter would extend vector ${\varvec{w}}$ into a support domain that is twice as long as the original distribution support domain, i.e. doubling the ${p}_{X}$. distribution support length, $\left(2n-1\right)\cdot \Delta x\approx 2n\cdot \Delta x=2{X}_{L}$, compared to the original distribution support length $n\cdot \Delta x={X}_{L}$, where $\Delta x$ is the constant length of each discrete distribution bin. In other words, convolution may convect distribution tail attributes farther "downstream" or deeper inside the tail.

In Eq. (14), values of ${\varvec{u}}$ and ${\varvec{v}}$ outside the length of the vector are constrained to be zero; however, this is not the case in practice. Consequently, later in this research, vectors ${\varvec{u}}$ and ${\varvec{v}}$ will be extrapolated linearly on the logarithmic scale; see the next section, in which ${\varvec{u}}$ the probability distribution function will represent ${\varvec{u}}$ and ${\varvec{v}}$ by, ${f}_{{X}_{1}}$, and ${\varvec{w}}$ by ${f}_{X}$.

Note that ${\varvec{w}}=\left(w\left(1\right),\ldots,w\left(n\right)\right)$ is a discrete representation of the empirical target distribution, ${p}_{X}$ from “Results” section, with $n$ denoting the length of distribution support $\left[0,{X}_{L}\right]$. For the sake of simplicity, this work is confined to the situation of one-sided positive valued random variables, i.e. $X\ge 0$.

In the remainder of this study, only deconvolution scenarios, i.e. ${\varvec{u}}={\varvec{v}}$ in Eq. (14), will be examined. According to Eq. (12), the distributions, ${p}_{X}$ and, ${p}_{{X}_{1}}$ will correspond to the vectors ${\varvec{w}}$ and ${\varvec{u}},$, respectively.

From Eq. (15) is evident that given ${\varvec{w}}=\left(w\left(1\right),\ldots,w\left(n\right)\right)$ one can successively locate unknown components ${\varvec{u}}={\varvec{v}}=\left(u\left(1\right),\ldots,u\left(n\right)\right)$, beginning with the first component $u\left(1\right)=\sqrt{w\left(1\right)}$, then the second $u\left(2\right)=\frac{w\left(2\right)}{2u\left(1\right)}$, and so on until $u\left(n\right)$.

As will be addressed further in this work, the authors propose simple linear extrapolation of self-deconvoluted vector $\left(u\left(1\right),\ldots,u\left(n\right)\right)$ towards $\left(u\left(n+1\right),\ldots,u\left(2n-1\right)\right)$. i\In other words ${p}_{{X}_{1}}$ will have its tail linearly extrapolated in the range $\left({X}_{L},2{X}_{L}\right).$ Note that ${p}_{{X}_{1}}$ can be called the deconvoluted distribution, that in discrete form is represented by the estimated vector ${\varvec{u}}$. Using Eq. (13) the original vector ${\varvec{w}}$ will be clearly extended and extrapolated into a support domain that is twice longer than the original distribution support domain, i.e. doubling the ${p}_{X}$ distribution support length $\left(2n-1\right)\cdot \Delta x\approx 2n\cdot \Delta x=2{X}_{L}$, relative to the original distribution support length $n\cdot \Delta x={X}_{L}$. To smooth out the tail of the original distribution, ${p}_{X}$, the authors conducted, ${p}_{X}$ tail interpolation, since the tail of the CDF distribution, is often rather regular for large tail values x. In particular, the NG extrapolation approach has been used; see “Methods” section. In addition, various nonlinear extrapolation techniques may be readily included in the suggested method; however, this would involve certain assumptions and biases.

## Results

This section provides numerical findings based on the deconvolution extrapolation approach presented. As stated in the Introduction, the deconvolution extrapolation method does not presuppose a particular functional class to extrapolate the distribution tail.

### Numerical results for exceedance probability distribution tail

Since in the majority of reliability analysis engineering applications, it is more important to estimate the probability of exceedance, i.e. 1CDF where CDF stands for cumulative density function than the marginal PDF, ${f}_{X}$ will represent the probability of exceedance 1—CDF in this paper, analogous to the marginal probability density function PDF, ${p}_{X}$ in “Results” section. However, the suggested strategy may be applicable to any monotonically declining concave or convex function tail that is sufficiently regular.

The "shorter" version of the original data set was extrapolated and compared to predictions based on the "longer" version to verify the recommended extrapolation strategy. Therefore, this study aims to demonstrate the efficacy of the recommended extrapolation approach by at least a few orders of magnitude.

Based on the preceding reasoning, one may implement an iterative approach, whereas in the marginal PDF, one can utilize 1-CDF and then construct a new artificially smoother CDF by integration. The latter may substantially improve the extrapolation process if distribution tail anomalies are present owing to a lack of underlying data.

Next, the process of discrete convolution, or rather de-convolution (since the objective was to determine the deconvoluted 1—CDF distribution, ${f}_{{X}_{1}}$, given the empirical distribution, ${f}_{X}$ is based on the sequential solution of Eq. (4). Since the deconvoluted values ${\varvec{u}}=\left(u\left(1\right),\ldots,u\left(n\right)\right)$ normally follow a monotonically diminishing trend (the same was expected for the empirical parent distribution ${f}_{X}$, it seems that certain final values of the resultant vector ${\varvec{u}}$, say, $\left(u\left(n-L\right),\ldots,u\left(n\right)\right)$ for some $L<n$. The latter is an unacceptable numerical inaccuracy since only positive numbers may represent distributions. To address this numerical difficulty, the following scaling method has been implemented^12,16^. As the pivot value, the lowest positive value ${f}_{L}$ of the specified distribution tail ${f}_{X}$ is used. The scaling is thus only a linear transition along the vertical y-axis of the logarithmic decimal scale.

15 $${g}_{X}= \mu \left({\text{log}}_{10}\left({ f}_{X}\right)-{\text{log}}_{10}\left({ f}_{L}\right)\right)+{\text{log}}_{10}\left({ f}_{L}\right)$$

with ${g}_{X}\left(x\right)$ being scaled ${\text{log}}_{10}$ version of the empirical base distribution ${f}_{X}$, with the reference level ${f}_{L}$ being intact. The scaling coefficient $\mu$ is conveniently chosen to avoid the occurrence of negative components in the resulting ${f}_{{X}_{1}}$. For both numerical examples studied in this paper, $\mu =1/3$ served that purpose well. Then when ${f}_{{X}_{1}}$ was found, and back convolution ${\widetilde{f}}_{X}={\text{conv}}\left({ f}_{{X}_{1}},{ f}_{{X}_{1}}\right)$ as in Eq. (13) was done, the inverse scaling with ${\mu }^{-1}$ was performed to restore the original scale, with ${\widetilde{f}}_{X}$ being extrapolated version of ${f}_{X}$.

### Synthetic example

This subsection studies the 3.65-day maximum wind speed process $X\left(t\right)$ simulated during the given time interval $\left[0, T\right]$. The underlying normalized non-dimensional stochastic processes $U\left(t\right)$ has been modelled as a stationary Gaussian process with zero mean value and a standard deviation equal to one. Therefore it was assumed that the $U\left(t\right)$ mean zero up-crossing rates satisfies the equality ${\nu }_{U}^{+}\left(0\right)={10}^{3}/T$, with $T=1$ year, the latter assumption is common in offshore wind engineering^20^.

For this numerical example, the «shorter» data record had been chosen to contain ${10}^{4}$ data points, which is equivalent to 100 years record, since wind speed maxima process $X\left(t\right)$ has 365/3.65=${10}^{2}$ data points per year; the full (the latter in this study referred to as «longer») data set had been chosen to contain ${10}^{6}$ data points (thus 100 times longer than the «shorter» data record) are equivalent to 10,000 years.

The underlying wind speed process $U\left(t\right)$ results in 3.65 days maximum analytical CDF distribution ${F}_{X}^{3d}\left(x\right)={\text{exp}}\left\{-q{\text{exp}}\left(-\frac{{x}^{2}}{2}\right)\right\}$ for the wind speed maxima process $X\left(t\right)$, with $q=10$. Note that under the above assumptions, the non-dimensional $X$-reference value ${x}^{100 {\text{yr}}}=4.80$, i.e. the wind speed with a return period of 100 years.

Note that artificial horizontal axis shift $x\to x-{x}_{\text{shift}}$ with ${x}_{\text{shift}}=1.5$ has been done in order to operate only with the distribution tail; in other words ${x}_{\text{shift}}={x}_{0}$ from Eq. (7) has been chosen as the cut-on tail marker. The latter makes an analytical estimate for the ${x}^{100 {\text{yr}}}=4.80-{ x}_{\text{shift}}=3.3$.

**Figure 2:**
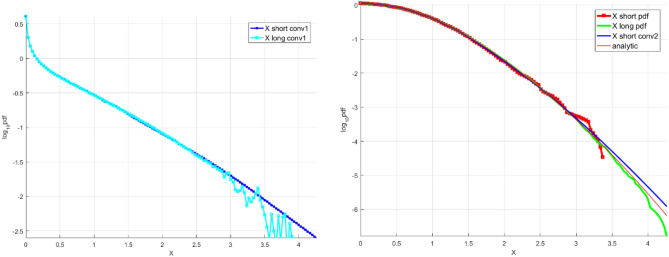
Synthetic wind speed data. Left: Scaled ${{\varvec{f}}}_{{{\varvec{X}}}_{1}}$ tail on the decimal log scale for the «shorter» data (cyan squares) «longer» data (blue squares). Right: Unscaled «shorter» decimal log scale ${{\varvec{f}}}_{{\varvec{X}}}$ tail, raw (red squares) and fitted (solid blue line, along with «longer» data (green line) and analytic (solid red line).

Figure 2 left presents synthetic wind speed data scaled results, Fig. 2 right presents the final unscaled results of the proposed in this paper technique, namely the «shorter» decimal log scale ${f}_{X}$ tail, extrapolated by deconvolution, along with «longer» data distribution tail and analytic distribution.

Finally, 100 independent numerical Monte Carlo (MC) runs identical to the one described above have been performed, i.e. both «shorter» and «longer» wind speed maxima data records were generated, with a subsequent comparison between the analytical 10,000 return period and the one estimated by the proposed deconvolution extrapolation. In addition, The Naess-Gaidai (NG) extrapolation technique has been performed for validation purposes. Validation using the above two methods suggested deconvolution extrapolation and taken as the benchmarking reference NG extrapolation technique has shown similar accuracy (within acceptable 5% relative deviation) in terms of 10,000 return period prediction.

Cross-validation or MC simulations are typically used to evaluate the performance of models in a robust manner^1^.

### Environmental example

It is generally known that the dynamics of wind speed is a highly nonlinear, multidimensional, and cross-correlated dynamic system that is always difficult to analyze. Moreover, the system dependability approach is crucial for buildings working in any particular region of interest and encountering genuine and sometimes harsh conditions. This section aims to demonstrate the efficacy of the previously mentioned technique by applying a novel method to the Norwegian maximum daily wind cast speeds data set in the vicinity of the Landvik wind station in southern Norway.

As five wind measurement sites, namely Landvik, Kjevik, Lindesnes Fyr, Lista Fyr, and Oksy Fyr, were selected for this research, their respective measured daily peak daily wind cast speeds were established as five environmental system components (dimensions) $X, Y, Z, \ldots$ therefore illustrating a five-dimensional (5D) environmental system.

During the 2010–2020 observation period, the extreme unidimensional response values for each of the five wind measurement sites were determined by doubling the maximum daily highest daily wind cast speed.

**Figure 3:**
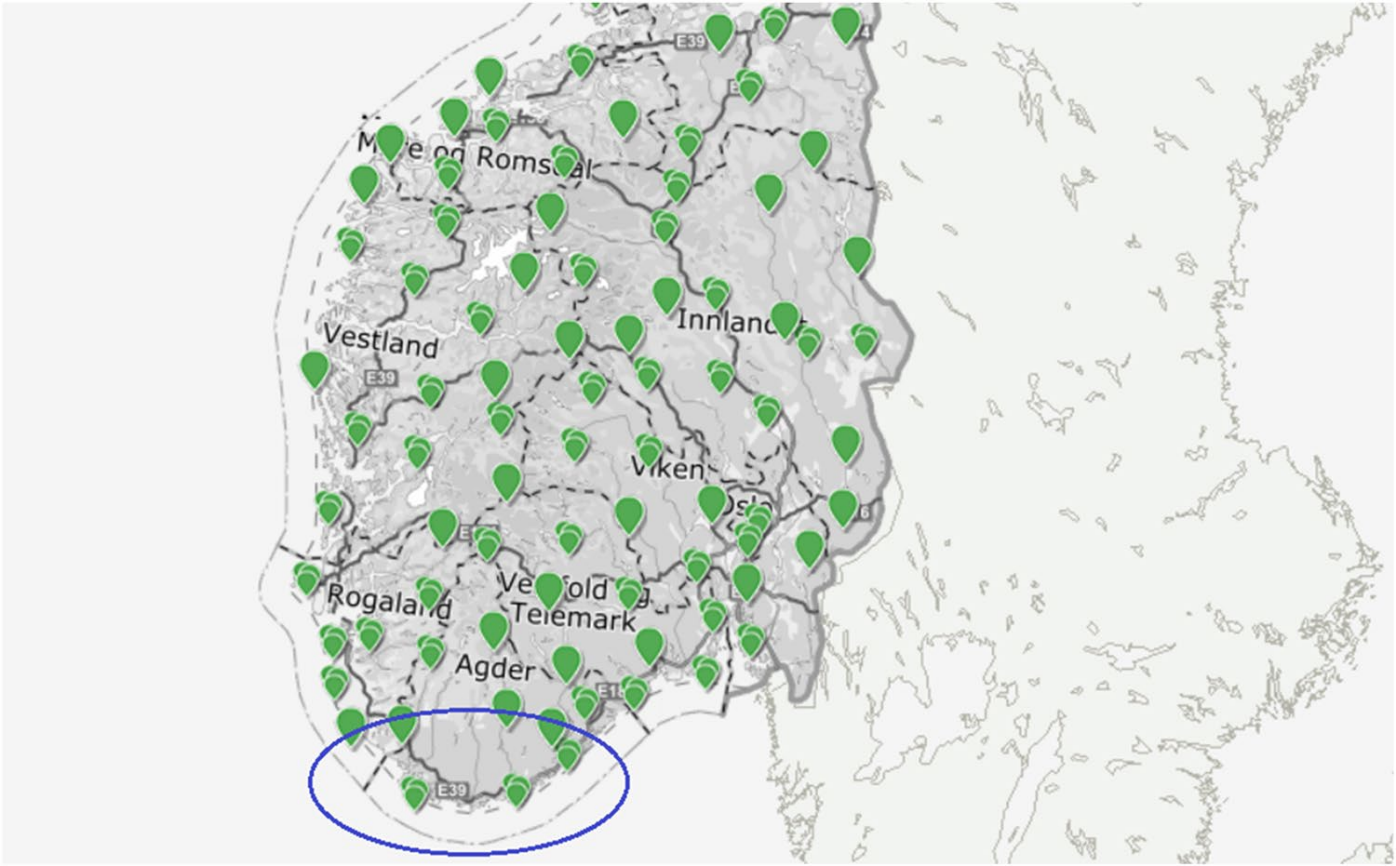
Wind speed measurements locations according to Norwegian Meteorological Institute^21^, a blue circle indicates the area of interest, https://seklima.met.no/.

**Figure 4:**
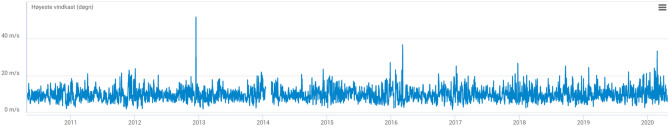
Daily largest highest daily wind cast speed at Landvik location during the years 2010 – 2020, Norwegian Meteorological Institute^21^.

Figure 3 presents wind measurement locations according to the Norwegian Meteorological Institute^21^; the blue circle indicates the area of interest. Daily largest highest daily wind cast speed at the Landvik location during the years 2010 – 2020 is shown in Fig. 4.

In order to unify all five measured time series $X, Y, Z$,... the following scaling was performed:

16 $$X\to \frac{X}{{\eta }_{X}}, Y\to \frac{Y}{{\eta }_{Y}}, Z\to \frac{Z}{{\eta }_{Z}}, \ldots$$

making all five responses non-dimensional and having the same failure limit equal to 1. Next, all local maxima from five measured time series were merged into one single time series by keeping them in time non-decreasing order: $\overrightarrow{R}=\left(\mathrm{max}\left\{{X}_{1},{Y}_{1},{Z}_{1}, \ldots \right\}, \ldots ,\mathrm{max}\left\{{X}_{N},{Y}_{N},{Z}_{N}, \ldots \right\}\right)$ with the whole vector $\overrightarrow{R}$ being sorted according to non-decreasing times of occurrence of these local maxima.

**Figure 5:**
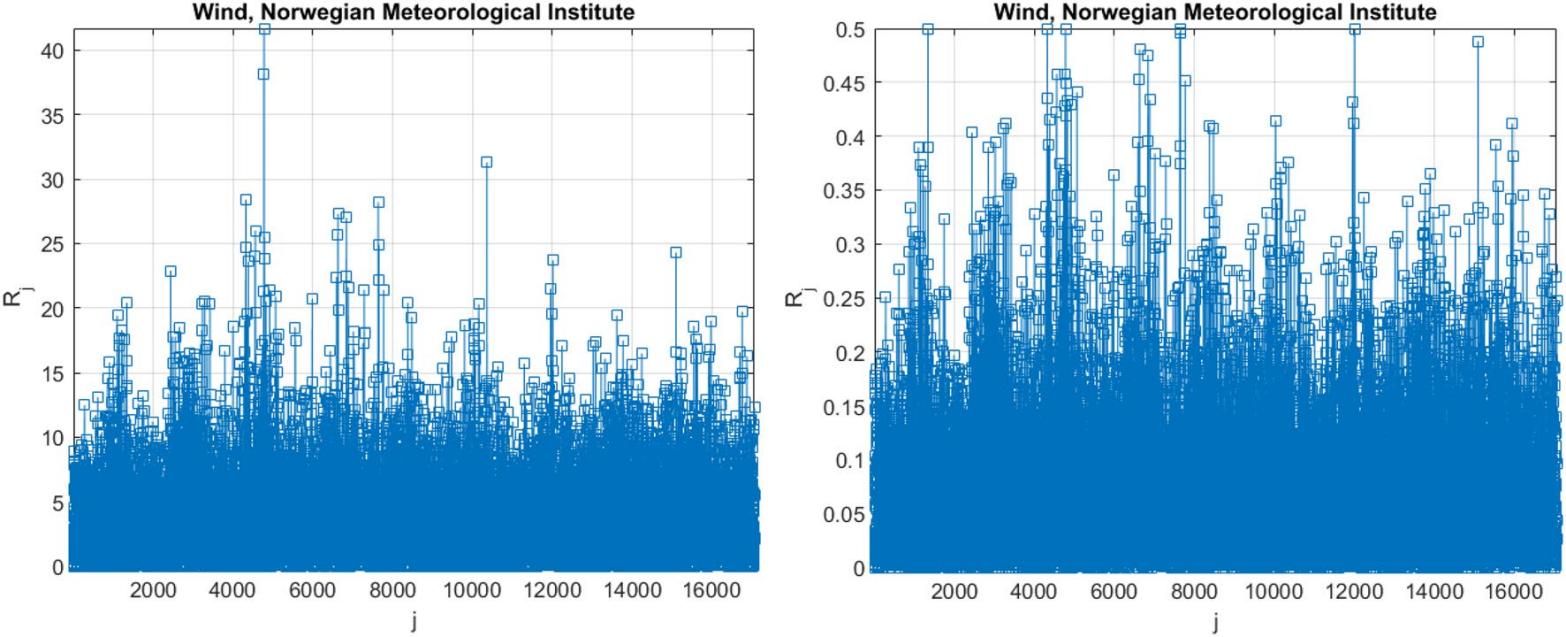
Left: unscaled raw daily largest highest daily wind cast speed data [m/sec], Right: scaled non-dimensional assembled 5D vector $\overrightarrow{R}$.

Figure 5 presents an example of a non-dimensional assembled vector $\overrightarrow{R}$, which is comprised of assembled local maxima of raw daily greatest highest daily wind cast speed data. The failure probability distribution tail was extrapolated towards a return time of 100 years. Note that vector, $\overrightarrow{R}$ has no physical significance on its own, since it is composed of wholly distinct response components. Index $j$ is only an index of local maxima encountered in a non-decreasing time sequence. Index $j$ is only an index of local maxima encountered in a non-decreasing time sequence. The «shorter» data record was constructed by extracting every tenth data point from the «longer» data record of wind speed.

**Figure 6:**
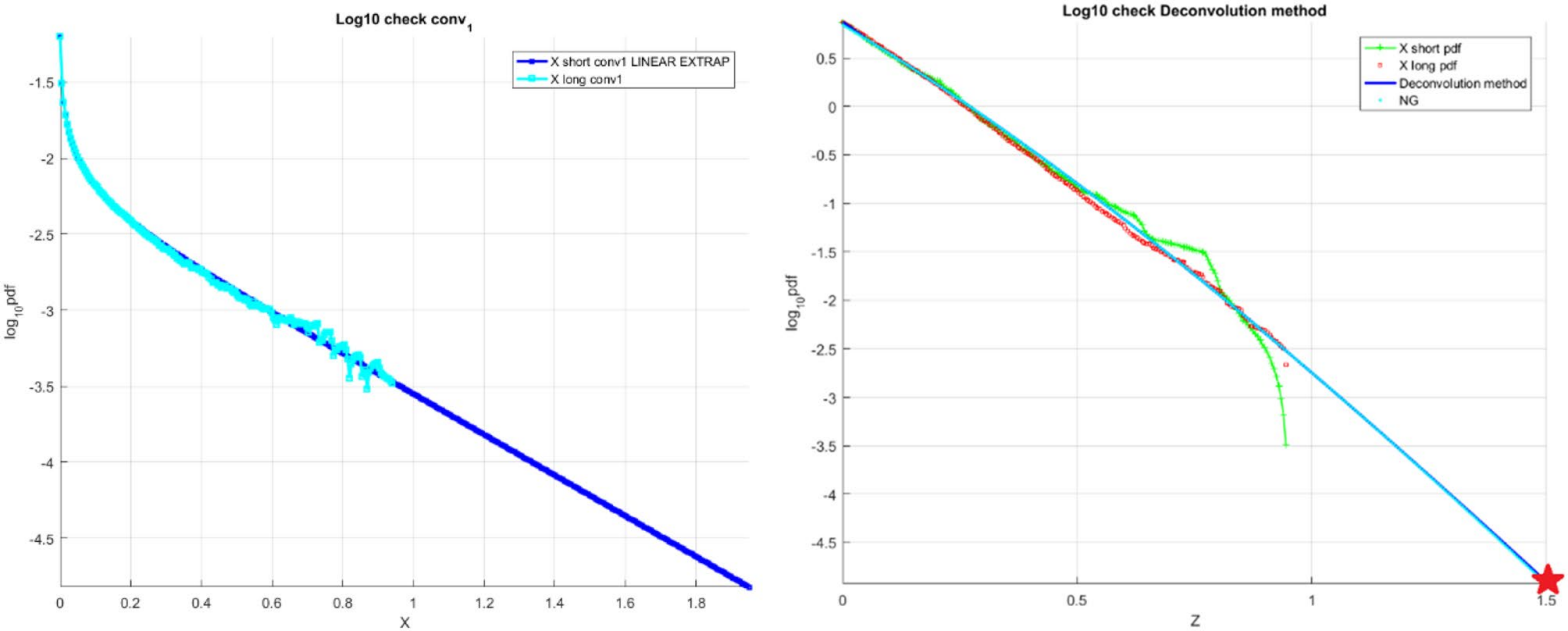
Wind speed data. Left: scaled ${{\varvec{f}}}_{{{\varvec{X}}}_{1}}$ tail on the decimal log scale for the «shorter» data (cyan), linearly extrapolated (dark blue). Right: unscaled raw «shorter» data (green) ${{\varvec{f}}}_{{\varvec{X}}}$ tail on the decimal log scale, extrapolated by the deconvolution method (dark blue), along with «longer» raw data (red) and NG extrapolation (cyan). Star indicates 100 years return period prediction.

Figure 6 on the left shows the «shorter» data record, ${f}_{{X}_{1}}$ tail, derived by deconvolution as in Eq. (13), and then linearly extrapolated in the terminal tail section to encompass the ${X}_{1}$ range corresponding to the «longer» data record. Figure 6 on the right displays the unscaled final outcomes of the approach suggested in this research, namely the «shorter» decimal log scale, ${f}_{X}$ tail, extrapolated by deconvolution, together with the «longer» data distribution tail and NG extrapolation.

It is seen that NG prediction matches the deconvolution method pretty well. Figure 6 on the right demonstrates that the suggested technique works pretty well, is based on the "shorter" data set and produces distributions that are relatively similar to those derived from the "longer" data set. Figure 6 on the right illustrates extrapolation according to Eq. (9) as well as a unique deconvolution approach from Sect. 0 towards a failure state with a 100-year return time, which is 1 and a bit beyond; $\lambda =0.05$ was chosen as the cut-on value. The dashed lines represent the extended 95% confidence interval according to Eq. (10). According to Eq. (5), $p\left(\lambda \right)$ is exactly proportional to the goal failure probability 1—P. (1). In accordance with Eq. (5), the system failure probability $1-P\approx {1-P}_{k}\left(1\right)$ may be computed. Note that in Eq. (5), $N$ is the total number of local maxima in the response vector $\overrightarrow{R}$. Due to convergence with respect to $k$ (see Eq. 6), it was determined that $k=5$ was an adequate conditioning value (6).

Note that, although innovative, the above-described technique has the evident benefit of using existing measured data sets rather effectively, owing to its capacity to handle system multidimensionality and conduct correct extrapolation using relatively small data sets.

## Conclusions

Classical dependability approaches for time series cannot properly handle systems with high dimensionality and cross-correlation between various system responses. The presented approach's primary advantage is its ability to examine the dependability of high-dimensional nonlinear dynamic systems.

This article examined the Norwegian greatest daily wind cast speeds obtained at two distinct wind speed measurement locations near the Landvik wind station from 2012 to 2022.

The techniques presented in this study have been previously confirmed by application to a variety of simulation models, albeit only for one-dimensional system responses, and highly accurate predictions have been achieved in general. This research attempted to establish a multidimensional dependability approach that was simple, robust, and applicable to a variety of applications.

It is possible to analyze both observed and numerically generated time series responses. It is shown that the suggested strategy generated an acceptable confidence interval. Consequently, the proposed technique may become useful for various nonlinear dynamic system dependability investigations.

In the case of Monte Carlo-type numerical simulation, unlike existing dependability approaches, the novel method does not need to restart numerical simulation each time the system fails. It is possible to analyze both observed and numerically generated time series responses.

Finally, the recommended technique applies to a vast array of technical applications. The offered example of naval architecture does not restrict the scope of application for the proposed technique.

## Data Availability

Meteorological data used in this paper is publicly available at the Norwegian Meteorological Institute, https://seklima.met.no/.
